# Supplementary material for: DeepViral: prediction of novel virus–host interactions from protein sequences and infectious disease phenotypes
Source: Bioinformatics. 2021 Mar 3;37(17):2722–9. doi: 10.1093/bioinformatics/btab147 (PMC8428617; doi:10.1093/bioinformatics/btab147)
Supplement: btab147_Supplementary_Data [file btab147_supplementary_data.pdf]

Supplementary materials: DeepViral: prediction of novel virus–host interactions from protein sequences and infectious disease phenotypes

Wang Liu-Wei[1], Şenay Kafkas[2], Jun Chen[1], Nicholas Dimonaco[4], Jesper Tegnér[1,3] and Robert Hoehndorf[1,2,*]

[1] Computer, Electrical and Mathematical Sciences and Engineering Division, King Abdullah University of Science and Technology, Thuwal 23955, Saudi Arabia,
[2] Computational Bioscience Research Center, King Abdullah University of Science and Technology, Thuwal 23955, Saudi Arabia,
[3] Biological and Environmental Science and Engineering Division, King Abdullah University of Science and Technology, Thuwal 23955, Saudi Arabia.
[4] Institute of Biological, Environmental and Rural Sciences, Aberystwyth University, Aberystwyth, Wales, UK

1 Comparison with existing methods

Method	SN (%)	SP (%)	ACC (%)	PPV (%)	NPV (%)	MCC	AUC	F1 (%)
DeNovo [Eid et al., 2016]	80.71	83.06	81.90	NA	NA	NA	NA	NA
VirusHostPPI [Zhou et al., 2018]	80.00	88.94	84.47	87.86	81.64	0.692	0.897	NA
Doc2Vec + RF [Yang et al., 2020]	**90.33**	96.17	93.23	95.99	90.74	0.866	**0.981**	93.07
RCNN [Chen et al., 2019]	89.88	95.58	92.73	95.38	90.46	0.857	0.974	92.52
DeepViral (seq)	89.36	96.89	93.13	96.68	90.13	0.865	0.960	92.86
DeepViral (seq + human embedding)	88.43	96.22	92.32	95.94	89.23	0.849	0.955	92.02
DeepViral (seq + viral embedding)	88.29	97.24	92.76	96.97	89.26	0.859	0.967	92.42
DeepViral (joint)	90.27	**97.58**	**93.91**	**97.43**	**90.93**	**0.881**	0.976	**93.68**

Table 1: Comparison with the state-of-the-art methods on the datasets of [Eid et al., 2016] (the performances of first 3 methods are from the original papers respectively). RCNN and the variants of DeepViral are evaluated 5 times independently to compute the mean of the metrics: SN - sensitivity, SP - specificity, ACC - accuracy, PPV - positive predictive value (precision), NPV - negative predictive value, MCC - Matthews correlation coefficient, AUC - area under the ROC curve. DeepViral (seq) only utilizes the protein sequences and the joint model also includes both the human and virus embeddings as input. The bold numbers represent the best metric for a dataset.

Implementation details: The dataset contains contains 5,020 positives and 4,734 negatives in the training set, and 425 positives and 425 negatives in the testing set. Since no validation set was used previously, we constructed a validation set by randomly sampling 10% of the training set, which was used for choosing the best epoch for RCNN and DeepViral. We truncated all longer sequences than 2,000 amino acids to only the first 2,000 for RCNN, due to the maximum sequence length limit of the model (similarly, first 1,000 amino acids for DeepViral). RCNN and DeepViral (seq) were implemented and evaluated for the entirety of the test set. For DeepViral variants with feature embeddings, a limited number of protein pairs, i.e. 2% of the test set, do not have relevant features available (some proteins are obsolete due to database updates) and thus are excluded from the test set. We evaluated both RCNN and the variants of DeepViral for 5 times and report the mean of the metrics.

2 Taxonomic information of Leave-One-Species-Out (LOSO) experiments

Family	Val/Test	Strain name	Taxon ID
Coronaviridae	Val	SARS-CoV	694009
	Test	SARS-CoV-2	2697049
Flaviviridae	Val	ZIKV	64320
	Test	ZIKV/H. sapiens/FrenchPolynesia/10087PF/2013	2043570
Orthomyxoviridae	Val	Influenza A virus (A/Hong Kong/156/97(H5N1))	130763
	Test	Influenza A virus (A/Vietnam/1194/2004(H5N1))	644788
Papillomaviridae	Val	Human papillomavirus type 16	333760
	Test	Human papillomavirus type 18	333761

Table 2: Taxonomic information of the LOSO experiments. Val - validation set, Test - test set. Taxon IDs are based on the NCBI Taxonomy Database [Sayers et al., 2009].

References

[Chen et al., 2019] Chen, M., Ju, C. J.-T., Zhou, G., Chen, X., Zhang, T., Chang, K.-W., Zaniolo, C., and Wang, W. (2019). Multifaceted protein–protein interaction prediction based on siamese residual rcnn. *Bioinformatics*, 35(14):i305–i314.

[Eid et al., 2016] Eid, F.-E., ElHefnawi, M., and Heath, L. S. (2016). Denovo: virus-host sequence-based protein–protein interaction prediction. *Bioinformatics*, 32(8):1144–1150.

[Sayers et al., 2009] Sayers, E. W., Barrett, T., Benson, D. A., Bryant, S. H., Canese, K., Chetvernin, V., Church, D. M., DiCuccio, M., Edgar, R., Federhen, S., Feolo, M., Geer, L. Y., Helmberg, W., Kapustin, Y., Landsman, D., Lipman, D. J., Madden, T. L., Maglott, D. R., Miller, V., Mizrachi, I., Ostell, J., Pruitt, K. D., Schuler, G. D., Sequeira, E., Sherry, S. T., Shumway, M., Sirotkin, K., Souvorov, A., Starchenko, G., Tatusova, T. A., Wagner, L., Yaschenko, E., and Ye, J. (2009). Database resources of the national center for biotechnology information. *Nucleic Acids Research*, 37(suppl_1):D5–D15.

[Yang et al., 2020] Yang, X., Yang, S., Li, Q., Wuchty, S., and Zhang, Z. (2020). Prediction of human-virus protein-protein interactions through a sequence embedding-based machine learning method. *Computational and structural biotechnology journal*, 18:153–161.

[Zhou et al., 2018] Zhou, X., Park, B., Choi, D., and Han, K. (2018). A generalized approach to predicting protein-protein interactions between virus and host. *BMC genomics*, 19(6):568.
